# Effectiveness of Empagliflozin With Vitamin D Supplementation in Peripheral Neuropathy in Type 2 Diabetic Patients

**DOI:** 10.7759/cureus.20208

**Published:** 2021-12-06

**Authors:** Sanjana Mehta, Parminder Nain, Bimal K Agrawal, Rajinder P Singh, Jaspreet Kaur, Sabyasachi Maity, Aniruddha Bhattacharjee, Jagannadha Peela, Shreya Nauhria, Samal Nauhria

**Affiliations:** 1 Department of Pharmacy Practice, Maharishi Markandeshwar College of Pharmacy, Maharishi Markandeshwar (Deemed to be University), Ambala, IND; 2 Department of Internal Medicine, Maharishi Markandeshwar Institute of Medical Science and Research, Maharishi Markandeshwar (Deemed to be University), Ambala, IND; 3 Department of Medicine, Pancham Hospital, Ludhiana, IND; 4 Department of Physiology, St. George's University School of Medicine, St. George's, GRD; 5 Department of Physiology, St. Matthew's University, George Town, CYM; 6 Department of Medical Genetics and Biochemistry, St. Matthew's University, George Town, CYM; 7 Department of Psychology, University of Leicester, Leicester, GBR; 8 Department of Pathology, St. Matthew's University, George Town, CYM

**Keywords:** empaglifozin, diabetes mellitus management, vitamin-d, peripheral neuropathies, type 2 diabets mellitus

## Abstract

Background: Neuropathy is the most prevalent broad-spectrum microvascular complication of diabetes. The present study aims to evaluate the effect of empagliflozin with vitamin D supplementation on diabetic peripheral neuropathy.

Methods: A prospective, randomized, controlled study was conducted for six months including 150 type 2 diabetic patients, divided into three groups (n=50/group): Group 1, patients on oral hypoglycemic agents; Group 2, patients on empagliflozin and Group 3, patients on empagliflozin with vitamin D. Biochemical parameters were estimated for outcome measurements and patients’ neuropathic pain was analysed using Douleur Neuropathique 4 Questions, Neuropathic Pain Symptom Inventory and Ipswich Touch the toes test questionnaire. Data were analysed using a one-way analysis of variance.

Results: Diabetic neuropathy in males was more prevalent (more than 50%) as compared to females in all three groups, with an average age of 50±6 years, along with a diabetic history of 15±4.5 years and a glycated hemoglobin A1C (HbA1C) level of >10%. The mean value of serum vitamin D level significantly increased by 64.7% (19±5 to 54±8 ng/mL; *p*<0.05). A remarkable decrease (by 17.4%) from baseline in the HbA1C level was observed after six months of treatment only in Group 3, whereas in other groups (1 and 2), there was a non-significant decrease in HbA1C levels when compared to baseline. Moreover, a significant improvement in neuropathic condition was seen only in Group 3.

Conclusion: The results indicated that empagliflozin with vitamin D supplementation significantly controlled or reduced HbA1C and improved diabetic neuropathic symptoms in patients. It is suggested that this combination can be considered as the primary therapeutic approach for neuropathic complications in diabetic patients.

## Introduction

Diabetes mellitus (DM) is the largest global epidemic of the 21st century and is associated with various complications classified as microvascular and macrovascular [1]. Microvascular complications include dysfunction of the nervous system (diabetic neuropathy), renal system (diabetic nephropathy), and ocular system (diabetic retinopathy). Macrovascular complications include cardiovascular disease, stroke, and peripheral vascular disease. The most prevalent and broad-spectrum microvascular complication with DM is neuropathy [2]. In neuropathy, there is loss of sensory function, where each level of the peripheral nerve is affected (peripheral neuropathy), i.e. the root of the distal axon, characterized by pain and numbness usually in hands and feet [3]. According to the latest figures by the International Diabetes Federation (IDF), 537 million adults (20-79 years old) worldwide have DM. The proportion of people with type 2 DM is increasing in most countries with a rise of 16% (74 million) since the previous IDF estimates in 2019. Approximately, 50% of diabetics tend to develop diabetic neuropathy [4]. It is well known that DM is a result of insulin resistance caused by an inflammatory state associated with elevated cytokine levels through which oxidative stress causes endothelial cell dysfunction [5]. Recently, there has been an evolution in understanding the pathophysiology of DM and related vitamin D deficiency [6]. Vitamin D controls DM by lowering the C-reactive protein (CRP) levels and reducing insulin resistance along with preventing cardiovascular abnormalities by improving endothelial function. Thus, vitamin D can possibly be a potential diabetes risk modifier [6].

Pre-clinical studies have demonstrated the association between low levels of neurotrophins (nerve growth factors [NGF]) with vitamin D deficiency [7]. Neurotrophins are necessary for calcium homeostasis along with the neuronal cell differentiation, development, survival, and functioning of both sensory and sympathetic neurons. A decline in neurotrophins causes defective neuronal response and imbalanced calcium homeostasis resulting in increased nerve damage by toxins [6,7]. Moreover, vitamin D deficiency worsens nerve damage, impairs nociception function, and lowers the threshold of pain. Vitamin D deficiency is prevalent in epidemic proportions worldwide and specifically all over the Asian and European continent with a prevalence of 70%-100% in the general population [7]. A review of the literature supported that vitamin D replacement therapy in addition to glucose-lowering diabetes treatment has a beneficial effect on the nervous system and muscle tissue [8]. Treating diabetic neuropathy has been a major challenge; even to date, various anti-diabetic drugs have been used for glycemic control along with diabetic complications, but they lack the protective effects on nerves and distal axons that scientists strive to achieve [9]. Empagliflozin (EMPA), which is from a new class of anti-diabetic drugs, i.e. sodium-glucose cotransporter-2 inhibitors (SGLT2i), has been found to prevent hypersensitivity and the loss of skin intraepidermal nerve fibers with mesangial matrix expansion in diabetic rats [10]. This study strives to evaluate the neuropathic improvement when EMPA is used in combination with vitamin D. Additionally, we attempted to identify a correlation between high serum levels of vitamin D and reduced neuropathic pain assessment outcomes in type 2 DM patients.

## Materials and methods

The present study was a cross-sectional, observational, open-label randomized controlled study of six-month duration, which evaluated the therapeutic effect of a new class of anti-diabetic drug, i.e. SGLT2i with vitamin D on diabetic neuropathic pain in type 2 DM patients (diabetic neuropathy as a primary complication). Patients from the Department of Medicine (outpatient and inpatient) in a tertiary care hospital were selected and analysed by three different neuropathic pain assessment scales, i.e. Douleur Neuropathique 4 Questions (DN-4), Neuropathic Pain Symptom Inventory (NPSI), and Ipswich Touch the toes test (ITTT) between September 2020 and August 2021 (including patient recruitment time). The institutional ethics committee (IEC) approval was obtained (MMDU/MMIMSR/IEC/1565) prior to conducting the study. Written informed consent was signed by all the patients for participation in the study.

Study design

The sample size was calculated based on the data obtained from previous studies using Power and Precision software (Biostat, Englewood, NJ). A total of 150 patients 41-60 years of age were identified according to inclusion and exclusion criteria and were divided into three groups (n=50/group) by simple randomization as Group 1, patients on oral hypoglycemic agents; Group 2, patients on EMPA 25 mg once daily and Group 3, patients on EMPA 25 mg once daily with vitamin D (60,000 IU/week). The data were normally distributed according to the Kolmogorov-Smirnov normality test. The parametric data were analysed using paired Student's t-test followed by Dunnett's test; *p*<0.05 was considered statistically significant. During the study, the drugs were prescribed as per guidelines, and patients were instructed not to change their medicines, diet, and lifestyle without the physician’s advice, throughout the study period.

Inclusion criteria were as follows: patients (aged 40-60 years) having type 2 DM for at least 10 years, taking one or two oral hypoglycemic agents regularly, with glycated hemoglobin A1C (HbA1C) >10%, low vitamin D levels (≤20 ng/mL), never on SGLT2i before inclusion in the study and having a DN-4 score more than 4. Patients with other types of diabetes, current or previous use of vitamin D or multivitamins, pregnant women, patients with chronic kidney disease stage 5, diabetic ketoacidosis, liver cirrhosis, hypothyroidism/hyperthyroidism, osteoporosis, patients already taking neuropathic drugs, anti-epileptics and patients with a body mass index more than 30 kg/m^2^ were excluded from the study.

The DN-4 questionnaire was used as a diagnostic tool to identify and assess polyneuropathy in diabetic patients; this questionnaire consists of two parts, i.e. interview of patients and examination of patients (which further comprises 10 items). Initial seven items were related to the patient’s personal complaints including sensational variations and pain; however, the last three items were linked with the physical examination. For comparison and analysis, the scoring system was based on “Yes” and “No” scored as 1 and 0 for each answer, respectively. The score of the questionnaire was in the range 0-10, where 0 was the minimum and 10 the maximum score. If a patient had a DN-4 score of less than 4, then the pain was unlikely to be neuropathic pain, but if the score was more than 4, then the pain was likely to be neuropathic pain [11].

To assess and characterize the nature of neuropathic pain, a self-rated questionnaire, NPSI, was taken into consideration that included 12 items grouped into five domains including superficial burning pain (Q1), deep pressing pain (Q2-Q3), paroxysmal pain (Q5-Q6), evoked pain (Q8-Q10) and paresthesia/dysesthesia (Q11-Q12). These items were rated on an 11-point numeric rating scale from 0 to 10 where 0 corresponds to “no symptom” and 10 signifies “worst symptom imaginable.” Moreover, this questionnaire consisted of two temporal items (Q4 and Q7) to assess the number of pain paroxysms and pain duration (assessed on a 5-point categorical scale, i.e. 1 to 5) where Q4 was assessed as 1 = “permanently,” 2 = “between 8 and 12 hours,” 3 = “between 4 and 7 hours,” 4 = “between 1 and 3 hours,” 5 = “less than 1 hour”, referred in the past 24 hours, and Q7 was assessed as 1 = “more than 20 hours,” 2 = “between 11 and 20 hours,” 3 = “between 6 and 10 hours,” 4 = “between 1 and 5 hours,” 5 = “no pain attack” [12].

To assess the sensitivity in the feet, a quick and easy ITTT was used based on a non-throbbing feather-like soft touch for one to two seconds to each adjacent foot toe, i.e. the sensitivity was checked for adjacent six toes of both the feet of the patient. If the touch was not felt, it was recorded as “N” and if the touch was felt, it was recorded as “Y”. If a patient felt touch in all six or five out of the adjacent six toes (both feet), then his/her sensation was considered normal and if the patient did not feel when touched at two or more out of the adjacent six toes, then he/she was considered likely to have reduced sensation and might be at risk of a diabetic foot ulcer [13].

Along with above-mentioned non-invasive tests, all other routine biochemical tests like liver function test (LFT), renal function test (RFT), HbA1C and serum vitamin D level tests were performed on all enrolled patients on the day of enrollment (day 1) and at the end of the study (after six months). All information related to patient demographics, medical history and response to the above questionnaires was recorded in the case report form.

## Results

A total of 150 type 2 DM patients were divided into three groups (as per the study design); the majority of patients affected from diabetes were males (more than 50%) as compared to females in all three groups. The mean age of patients was 50±6.2 years and the body mass index (BMI) range of patients was 26-30 (27.7±2.1) kg/m^2^. All the patients had type 2 DM for more than 10 years. The patients were recruited from inpatient (14±2%) and outpatient (86±4%) wards for all three groups. There was no statistical difference found in demographic data of type 2 DM patients in all three groups (Table 1).

**Table 1:** Demographic data of the study population (n=150) BMI = body mass index, DM = diabetes mellitus

Overall	Demographic data	Group 1 (n=50)	Group 2 (n=50)	Group 3 (n=50)
Age (years)	41-50	46% (n=23)	48% (n=24)	52% (n=26)
	51-60	54% (n=27)	52% (n=26)	48% (n=24)
Gender	Male	58% (n=29)	60% (n=30)	54% (n=27)
	Female	42% (n=21)	40% (n=20)	46% (n=23)
BMI (kg/m^2^)		26.9±0.5	28.9±0.7	27.2±0.8
Duration of type 2 DM (years)	10-15	44% (n=22)	42% (n=21)	46% (n=23)
	15-20	56% (n=28)	58% (n=29)	54% (n=27)
Type of patient	Inpatient	12% (n=6)	16% (n=8)	14% (n=7)
	Outpatient	88% (n=44)	84% (n=42)	86% (n=43)

Drug-prescribing patterns in recruited patients were studied and analysed on the basis of symptoms and existing co-morbid conditions. The major categories of drugs prescribed were oral hypoglycemic agents (monotherapy or dual therapy) along with anti-hypertensive, anti-platelets, statins, and antibiotics.

All the parameters under investigation such as bilirubin direct, bilirubin total, serum glutamic oxaloacetic transaminase (SGOT), serum glutamic pyruvic transaminase (SGPT), alkaline phosphatase (ALP), urea, creatinine, uric acid, sodium, and potassium levels were found in the normal range among all three groups. The mean baseline serum vitamin D levels in all the three groups of type 2 DM patients at the time of inclusion were 17.78±5.3, 18±4.1, and 18.91±5.3 ng/mL, respectively. But the levels of vitamin D were significantly increased to 53.71±7.8 ng/mL in Group 3 (*p*<0.05) after giving vitamin D supplementation for six months. The serum vitamin D level significantly (*p*<0.05) increased by 64.7% (18.91±5.3 to 53.71±7.8 ng/mL) in Group 3 after giving vitamin D supplementation for six months. Moreover, HbA1C levels remarkably decreased from 13.09±2.0% to 10.80±2.8% only in Group 3 after six months of supplementation with respect to baseline parameters. Table 2 shows a comparison of biochemical parameters of the study population.

**Table 2:** Comparison of biochemical parameters of the study population SGOT = serum glutamic oxaloacetic transaminase, SGPT = serum glutamic pyruvic transaminase, ALP = alkaline phosphatase, HbA1C = glycated hemoglobin A1C *p<0.05 was considered as significant when compared with baseline data of the same group.

S. no.	Biochemical parameters	Group 1 (n=50)		Group 2 (n=50)		Group 3 (n=50)	
		Baseline values	Values after 6 months (mean±SD)	Baseline values	Values after 6 months (mean±SD)	Baseline values	Values after 6 months (mean±SD)
1	Billirubin direct (mg/dL)	1.33±0.2	1.21±0.5	1.70±0.4	1.61±0.7	1.49±0.6	1.30±0.5
2	Billirubin total (mg/dL)	1.741±0.4	1.69±0.6	1.19±0.3	1.10±0.7	1.36±0.5	1.28±0.3
3	SGOT (units per liter of serum)	62.91±5.2	62.32±3.6	60.13±3.9	61.20±4.8	61.44±3.7	57.91±2.6
4	SGPT (units per liter of serum)	73.6±4.6	69.61±3.7	76.8±5.3	68.27±4.9	79.86±4.1	72.79±5.0
5	ALP (IU/L)	149.4±14.6	142.5±13.6	131.33±9.5	129.6±10.6	138.4±11.4	120.35±13.5
6	Urea (mg/dL)	50.48±13.6	50.13±12.4	46.93±12.2	44.19±9.6	51.5±15.0	45.49±10.9
7	Creatinine (mg/dL)	2.01±0.8	2.16±1.1	2.07±1.1	1.94±1.2	1.99±0.9	1.82±1.8
8	Uric acid (mg/dL)	4.16±1.6	4.06±1.7	4.72±1.4	4.21±1.5	4.9±1.1	4.37±1.6
9	Sodium (mEq/L)	137.1±13	136.4±11	131.73±18	135.57±14	132.22±20	130.69±15
10	Potassium (mmol/L)	4.44±0.7	4.26±0.9	4.05±1.1	4.1±1.0	4.23±0.8	4.3±1.1
11	Vitamin D (ng/mL)	17.78±5.3	17.73±6.2	18±4.1	19.98±5.3	18.91±5.3	53.71±7.8*
12	HbA1C	13.78±2.9	13.21±2.1	14.04±3.1	13.11±1.9	13.90±2.0	10.80±2.8

**Figure 1:**
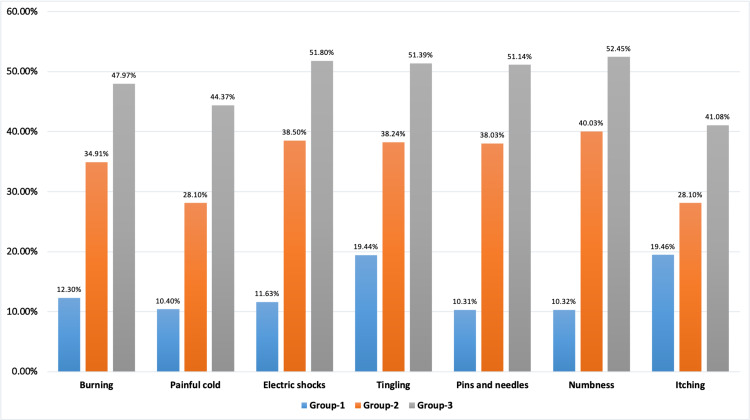
Percentage of improvement in domains of the Douleur Neuropathique 4 Questions (DN-4) score

Patients were included on the basis of the DN-4 score (those with a score of more than 4 were considered to have the pain of neuropathic origin). Patients were asked to define their pain in seven domains: burning, painful cold, electric shock, tingling, feeling of pins and needles, numbness, and itching. Results of our study showed that initially, the percentage of patients experiencing the seven domains as per the DN-4 questionnaire was higher in all three groups: burning, >52.7%; painful cold, >37.5%; electric shock, >72.1%; tingling, >83.2%; feeling of pins and needles, >63.69%; numbness, >62.3%; and itching, >43.1%. After receiving treatment as per study methodology, there was a maximum change in the percentage of patients only in Group 3: burning, 47.97%; painful cold, 44.37%; electric shock, 51.80%; tingling, 51.39%; feeling of pins and needles, 51.14%; numbness, 52.45%; and itching, 41.08% (Figure 1).

**Figure 2:**
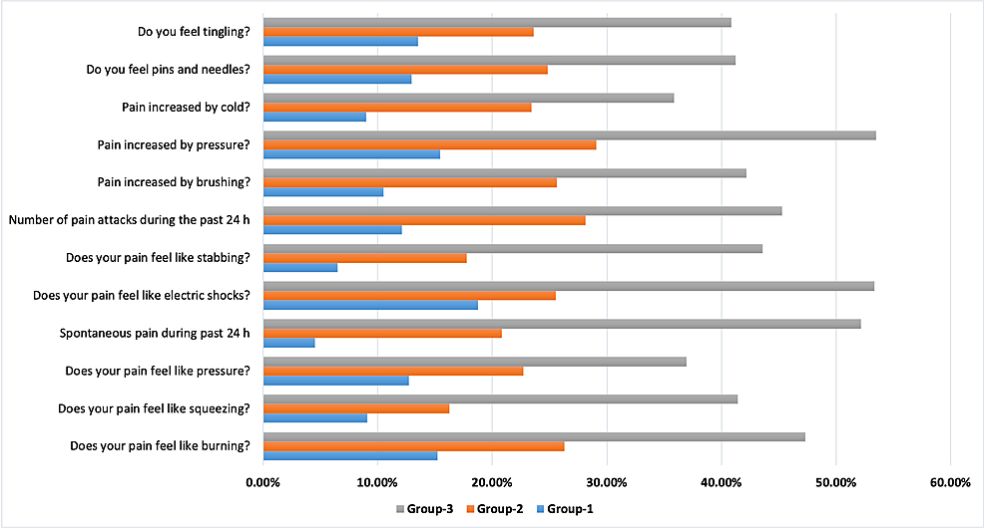
Percentage improvement in Neuropathic Pain Symptom Inventory (NPSI) scores

The assessment of the nature of neuropathic pain was done by the NPSI questionnaire. Results of the NPSI score showed that at the time of recruitment in the study, the domain score range in all the three groups varied: burning sensation (5.7% to 6%), squeezing sensation (4.3% to 4.5%), pressure (4.4% to 4.7%), spontaneous pain in the past 24 hours (2.2% to 2.4%), electric shock (4.5% to 4.8%), stabbing sensation (4.5% to 4.7%), number of panic attacks (3.3% to 3.4%), pain increased by brushing (3.8% to 4.1%), pain increased by pressure (4.2% to 4.6%), pain increased by cold (3.1% to 3.4%), feeling of pins and needles (5.4% to 5.7%) and tingling (5.5% to 5.9%). However, after giving treatment for six months, the percentage score decreased in Groups 1 and 2, but was non-significant. On the other hand, in Group 3, there was a significant (*p*<0.05) decrease in all the parameters of neuropathic pain and patients showed significant improvement in the percentage score: burning sensation (47.33%), squeezing sensation (41.44%), pressure (36.95%), spontaneous pain in past 24 hours (52.17%), electric shock (53.33%), stabbing sensation (43.61%), number of panic attacks (45.29%), pain increased by brushing (42.19%), pain increased by pressure (53.47%), pain increased by cold (35.88%), feeling of pins and needles (41.22%) and tingling (40.86%). Figure 2 shows the percentage improvement in NPSI scores.

**Figure 3:**
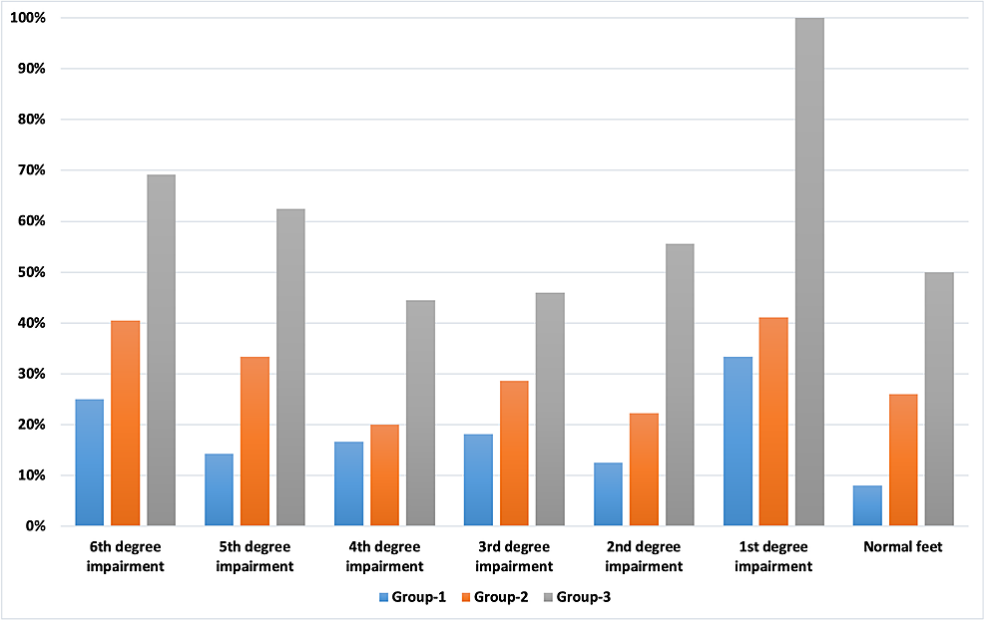
Percentage improvement score of the Ipswich Touch the toes test (ITTT)

ITTT was used to assess the sensitivity in diabetic neuropathic patients’ feet. Initially before treatment, the number of patients with 6th degree impairment (worst condition) was high as compared to patients with 5th, 4th, 3rd, 2nd and 1st degree impairment in all the three groups. Moreover, there was no patient with normal feet sensations in all three groups. The results after treatment with EMPA along with vitamin D (60,000 IU/week) showed a remarkable reduction in all the degrees of impairment and patients experienced an improvement in sensitivity along with prevention of foot ulcers on follow-up. All the patients (100%) recovered from 1st degree impairment and had normal feet sensation after receiving EMPA with vitamin D. Furthermore, the percentage of patients with 6th, 5th, 4th, 3rd and 2nd degree showed an impairment improvement by 69.23%, 62.50%, 55.55%, 46% and 44.44%, respectively. Figure 3 shows the percentage improvement score of ITTT.

## Discussion

An epidemic of diabetes in Asians is expected to rise over the next several years with India projected to have the largest diabetic population by 2030 [14]. The male-to-female ratio of type 2 DM neuropathic patients in our study suggests that neuropathy is more prevalent in diabetic males in India. These results are however similar to the WHO Expert Committee findings on diabetes that in 1980 reported a dominance of diabetic males among South Asian races [15]. Asians have several underlying risk factors such as increased insulin resistance, increased visceral adiposity despite a non-obese BMI, impaired β-cell function, an abnormal adipokine profile, and certain high-risk genetic polymorphisms that predispose them to have diabetes [16]. A higher than normal BMI was consistently associated with an increased probability of being diagnosed with type 2 DM. Similar results were found in our study where both men and women in the BMI range from 26.9±0.5 to 28.9±0.7 kg/m^2^ for the overweight category were at an increased risk of developing diabetes-related complications [17]. Our BMI results are consistent with previous studies that have also concluded that excess weight and obesity were the major contributing factors to type 2 DM and its complications for both men and women [18].

A major problem in the treatment of DM is neuropathic pain. For ages, biguanides such as metformin have been the first choice of drug for DM either as monotherapy or as a combination of biguanides with sulphonylureas and other neurotonic medicines for the treatment of neuropathic complications, although sensory profiles are heterogeneous in neuropathic pain disorders, and subgroups of patients respond differently to treatments [19]. A new class of anti-diabetic drugs, i.e. SGLT2i, has been proven to improve nerve conduction with a reduction in neuropathic pain in diabetic rats (pre-clinical studies) [10]. The results of the present study show an improvement in the control of glycemic profile (HbA1C) as well as diabetic complications on combining EMPA with vitamin D. SGLT2i are found to be safe without any significant change in the biochemical parameters. Group 3 with EMPA and vitamin D achieved significant improvement in HbA1C (10.8 on average) compared to other groups (around 13). Thus, improved diabetes control itself improves neuropathy symptoms and our study results are consistent with this as Group 3 showed significant (*p*<0.05) improvement in neuropathic pain symptoms such as burning, painful cold, electric shock, tingling, feeling of pins and needles, numbness with itching, and also improvement in the degree of neuropathic impairment of feet.

In contrast, previous studies have shown that when diabetics are treated with metformin for a prolonged duration at higher doses, it decreases serum cobalamin, and increases levels of methylmalonic acid and homocysteine, thus becoming the leading cause of severe peripheral diabetic neuropathy [20]. This potentially gives rise to the need for combining neurotonic and other supplements to be given with other oral hypoglycemic agents to prevent diabetic neuropathy [21]. EMPA was shown to gradually reduce the diabetic HbA1C levels and neuropathic complaints in our study and to have a neuroprotective effect in pre-clinical settings [22]. Thus, it could be considered as a drug of choice for diabetic patients with diabetic neuropathic complications.

## Conclusions

In summary, we determined whether EMPA in combination with vitamin D exerted a therapeutic effect beyond glycemic control on neuropathic complications in DM. The combination ameliorated neuropathy as well as systemic oxidative stress while exerting minimal to absent side effects. To our knowledge, our study is one of the first to present data that supports the use of EMPA in combination with vitamin D in the reduction of diabetic neuropathy while improving patients' quality of life. In the future, it can potentially be used as a first-line treatment in diabetic neuropathic patients. This study also supports the view that the concept of combination therapies can pave the path towards more individualized, patient-specific treatments.
